# Youth, Caregiver, and Prescriber Experiences of Antipsychotic-Related Weight Gain

**DOI:** 10.1155/2013/390130

**Published:** 2013-11-20

**Authors:** Andrea Lynn Murphy, David Martin Gardner, Steve Kisely, Charmaine Cooke, Stanley Paul Kutcher, Jean Hughes

**Affiliations:** ^1^College of Pharmacy and Department of Psychiatry, Dalhousie University, 5968 College Street, P.O. Box 15000, Halifax, NS, Canada B3H 4R2; ^2^Department of Psychiatry and College of Pharmacy, Dalhousie University, QEII HSC, AJLB 7517, 5909 Veterans' Memorial Lane, Halifax, NS, Canada B3H 2E2; ^3^School of Population Health, University of Queensland, Herston, QLD 4006, Australia; ^4^Department of Health and Wellness, Barrington Tower, P.O. Box 488, Halifax, NS, Canada B3J 2R8; ^5^Dalhousie University, Department of Psychiatry, Dalhousie University/IWK Health Centre 5850 University Avenue, P.O. Box 9700, Halifax, NS, Canada B3K 6R8; ^6^School of Nursing, Dalhousie University, 5869 University Avenue, P.O. Box 15000, Halifax, NS, Canada B3H 4R2

## Abstract

*Objectives*. To explore the lived experience of youth, caregivers, and prescribers with antipsychotic medications. *Design*. We conducted a qualitative interpretive phenomenology study. Youth aged 11 to 25 with recent experience taking antipsychotics, the caregivers of youth taking antipsychotics, and the prescribers of antipsychotics were recruited. *Subjects*. Eighteen youth, 10 caregivers (parents), and 11 prescribers participated. *Results*. Eleven of 18 youth, six of ten parents, and all prescribers discussed antipsychotic-related weight gain. Participants were attuned to the numeric weight changes usually measured in pounds. Significant discussions occurred around weight changes in the context of body image, adherence and persistence, managing weight increases, and metabolic effects. These concepts were often inextricably linked but maintained the significance as separate issues. Participants discussed tradeoffs regarding the perceived benefits and risks of weight gain, often with uncertainty and inadequate information regarding the short- and long-term consequences. *Conclusion*. Antipsychotic-related weight gain in youth influences body image and weight management strategies and impacts treatment courses with respect to adherence and persistence. In our study, the experience of monitoring for weight and metabolic changes was primarily reactive in nature. Participants expressed ambiguity regarding the short- and long-term consequences of weight and metabolic changes.

## 1. Introduction

Second generation antipsychotic prescribing to young people under 25 years of age is increasing internationally [1–12]. The rise in prescription trends has generated controversy given available pediatric evidence for second generation antipsychotic effectiveness and safety data and the unknown long-term consequences with intermittent or continuous exposure [13–16]. Antipsychotic-related weight gain and changes in the metabolic profile (e.g., glucose and lipid homeostasis) that occur following, or in concert with, weight gain are frequently discussed as significant treatment-related issues. The explicit mechanisms of antipsychotic-related weight gain are not fully understood [17–24] nor are the best management approaches (e.g., lifestyle, pharmacologic), but research in these areas continues [17, 25–33].

 Predictive or risk factors for antipsychotic-related weight gain are also unknown but some inferences can be made from available syntheses of the literature. These factors include lower pretreatment body mass index (BMI), triglyceride levels, more negative symptoms, polypharmacy, concomitant antidepressants, and younger age [34, 35]. Predisposing factors are similar for pediatrics and antipsychotic-naïve patients, and younger age groups appear to be at highest risk [36, 37]. Weight gain may also be more dramatic with younger patients compared to adults [36].

Given the local and international trends in utilization and controversies regarding effectiveness and safety of antipsychotics, we explored the lived experience of youth taking antipsychotics, their caregivers, and prescribers through a qualitative study using an interpretive phenomenological approach. This paper focuses on the experience of weight gain for youth taking antipsychotics, their caregivers, and the prescribers.

## 2. Methods

To understand the experience of antipsychotic use, we used interpretive phenomenology. Phenomenology studies the person in the situation, rather than in isolation [38]. We followed a staged approach modeled after Diekelmann et al. [39]. In stage one, all transcripts were read by AM and DG as a whole to gain a complete picture or overall understanding of the data considering weight gain. In stage two, we summarized sections of the transcripts and identified categories within weight gain. We met weekly for a month to discuss our interpretations of the categories and provided text quotes to demonstrate support. We worked to achieve consensus in the categories. In stage three, we continued with independent analysis of transcripts and compared interpretations of the categories with supporting text. In stage four, we sought to examine relational themes. Throughout the process we engaged in reflexivity through journaling. Among other strategies, our analysis process facilitated achieving credibility and dependability. We used several approaches for confirmability and transferability. Transferability may be limited to an extent as the study occurred within a Canadian province with a predominantly publically financed system (70% public, 30% private).

## 3. Sample and Recruitment Procedures

We recruited low income youth, defined as youth receiving government income assistance, aged 11 to 25 years, who were prescribed antipsychotics within the last two years. We also recruited their caregivers/support people. General practitioners and psychiatrists who prescribed antipsychotics to youth in the last six months and licensed to practice in Nova Scotia, a Canadian province, were eligible and were informed of the study for physician recruitment purposes and also to facilitate youth and caregiver recruitment. We advertised broadly in public places and on the Internet. All participants were offered the choice of a one time interview or focus group, and each participant was paid a one-time honorarium. 

## 4. Data Collection

Separate interview guides (available upon request) with main and probing questions were developed for each group of participants. Youth, caregiver, and physician interviews and one physician focus group were conducted between January of 2010 and August of 2010. All interactions with participants were recorded and transcribed except one face-to-face interview (one youth refused to be recorded). All audio recordings were transcribed, and, during cleaning of transcripts by the interviewer, field notes and jottings were used to insert comments and reflections when appropriate. Responsive interviewing techniques were used with an iterative process of data analysis occurring during data collection [40]. Once cleaned, transcripts were stored and organized using Atlas.ti 6.2.

### 4.1. Ethical Approvals

Ethics approval was obtained from the research ethics boards (REB) of Capital District Health Authority and the IWK Health Centre given that our population spanned young people and adults. Both of these REBs have reciprocal ethical agreements with Dalhousie University REB.

### 4.2. Findings

The theme of weight gain emerged from an analysis of approximately 13 hours of audio from 18 youths who ranged in age from 13 to 26.  Table 1 shows the range of medications and diagnoses as described by participants. The two 26-year olds were included in the analysis as their most recent antipsychotic exposures were in their 25th year of life. One youth and caregiver pair were related (i.e., mother and son) in our study, and they were interviewed separately. We collected 11 audio hours from 10 female caregivers, who will herein be referred to as “parents” given all caregivers described themselves as a “parent” and 7 audio hours from 11 prescribers (*n* = 5 prescribers in a focus group and *n* = 6 prescriber individual interviews).

Eleven of 18 youth, six of ten parents, and all prescribers discussed antipsychotic-related weight gain. Participants were highly attuned to the number in pounds as a metric for weight changes. Within the theme of weight gain, we further categorized and coded the data with body image, adherence and persistence, managing weight gain, and metabolic effects. Within the experiences of participants, these concepts were often inextricably linked, but each maintained significance as a separate concept.

### 4.3. Body Image

Changes in body weight affected body image and the potential to experience stigma related to weight gain. Youth were not only coping with their own emotions with weight changes but also managing the responses of others to their physical appearance. Parents and prescribers similarly discussed the concept of body image, but the concerns more shifted towards long-term implications of weight gain from a safety lens or adherence to medications. Prescribers' perceptions of the youth consciousness with weight changes were an underlying consideration in the prescriber choice of antipsychotic and anticipated adherence patterns in youth.

*Youth 9:*

* “I don't like that I have a belly. People are like, ‘Oh, you got chubby in the face.' And I'm like, ‘It's because I'm taking my medication, bastard. Shut the f%$# up!'." *

*Youth 11:*

*“… I've gained at least 15 pounds. And probably… I heard that usually there's a 6 month cap on it. So I'm in the clear, I guess. But I'm completely unhappy with my body.”*

### 4.4. Managing Weight Gain

Participants in our study discussed strategies that were used in mitigating weight gain or managing potential or existing weight gain through various choices. These included consideration of the initial choice of antipsychotic in terms of its potential to cause weight gain, switching of antipsychotics, lifestyle interventions (e.g., diet, exercise), pharmacological treatments, and nonadherence or eventual antipsychotic discontinuation. 

For youth, there was a frustration expressed when results of their efforts to minimize weight gain were not what they expected. Youth employed lifestyle strategies such as diet and exercise or talked about planning to initiate these strategies. Medication switches were discussed although these were initiated by prescribers. Discontinuation initiated by youth themselves was also used as an approach to stopping weight gain. 

One prescriber in the focus group was the only prescriber to discuss nonswitch pharmacologic options such as metformin to manage antipsychotic induced weight gain, and other members of the group appeared unaware of this option based on the ensuing discussion. For initial antipsychotic choice, several prescribers discussed their previous experiences with patients who had gained weight and also their own values as being influential in how they made their antipsychotic choices. 

Parents discussed youth engaging in behaviours to manage weight including exercising and dieting and also stopping medications. It was also evident from several parents that they made distinctions among antipsychotics, including “newer” versus “older” agents, about which were better or worse for causing weight gain, and revealing preferences for agent choice for their youth.

*Youth 7:*

*“… And I was exercising, eating the same way. I find taking weight off is impossible. Weight gain, weight gain, weight gain. Big time on the weight gain on the pill.”*

*Youth 13:*

*“Well, I had a lot of weight gain on the Risperdal. Interviewer: And is this one supposed to be better? Youth 13: Yes, it's supposed to be better and it has been because I've actually lost weight on it since…  … Interviewer: Can you put a number on what you think you've gained? Youth 13: Overall, it was like 100 pounds.”*

*Parent 7:*

*“… The Seroquel is not as bad as the Risperdal. … Yes. But the Seroquel has a weight gain thing too but it's not as bad for her anyway.”*

*Prescriber 4:*

*“And a situation where I have 2 parents who are [profession] [and] a child with autism. I started the child on the medication. The child started to gain weight. They were so desperate to keep him on medication because it changed his life, it changed their life. They did everything possible in the way of diet management, exercise management. Just everything possible to make sure that they minimized any metabolic effects.”*

*Prescriber 5:*

*“… If somebody told me, ‘Take this drug. It will make you feel better but you will gain 30 pounds,' I'd say, ‘no thanks'.” *

### 4.5. Adherence and Persistence

Adherence is described as the extent that patients follow instructions provided for treatments [41] including conforming to dosing, frequency, and timing of medications [42]. Persistence is defined as the length of time that a therapy is taken from initiation to discontinuation [42]. Medication adherence and persistence are considered important components in managing symptoms of mental illness. Participants in our study discussed adherence, nonadherence, and choices made with persistence to medication directly in the context of weight gain. It seems that youth who were committed to adhering to, and persisting with, their antipsychotics had realized or perceived a benefit from treatment or understood it to be important. Youth 7 discussed exercising in response to gaining “40 pounds in nine months” after he “agreed to take” his medication and also stating, “I've got to stay on this medication and keep taking it”. For one youth, she discussed discontinuing her antipsychotic due to sedation and weight gain and that this decision irreparably altered the patient-prescriber relationship.

Prescribers similarly discussed how weight gain would promote nonadherence, and this mainly centred on the belief that youth would be concerned with body image. Weight gain was generally seen as a barrier to adherence and a reason for nonadherence and lack of persistence. One prescriber's experience was that youth often withheld information about whether they persist with therapies. It also became evident that, for some prescribers, they too were challenged regarding adherence and persistence with intended treatment courses for their patient in light of weight gain. One prescriber shared information around ambivalence when weight gain occurs in the face of benefits.

*Youth 2:*

*“Like January/February 2008 when I stopped taking this medication, I went to him and I was like, ‘Look, I don't know why I'm taking this. It's a small dose but I'm tired all the time. I'm gaining weight.' And I'm like, ‘I've actually already stopped taking it.' And he's like, ‘Well, if you're not going to take the medication I prescribed you, there's nothing more I can do for you. I can have your files transferred to a different psychiatrist,' which is closer to where I live so I wouldn't have to go to [city] every time.” *

*Parent 8:*

*“… Then she started to gain some weight with the Invega. And my daughter is very petite and very, very conscious of her appearance and things like that.  … And gaining weight just wasn't in the cards. And she was really quite upset by that. … The barriers really are around the weight gain and then they don't want to stay on it.” *

*Focus Group*

*Prescriber 1:*

*“ So even though they may not be getting diabetic, the fact that they've gained 25 pounds on one of the antipsychotics, they may stop taking it. So that will impact. And they don't tell you.” *

*Prescriber 4:*

*“… Antipsychotic medications to some extent I have a little more concern about. And yet parents are a little less aware of that, unless they are well informed parents. Or until the child has been on the medication for 3 months and they start to see the child is gaining a bit more weight than they need. So we have to work to help them appreciate that this is important. But then we run into a different kind of ambivalence. The child might be functioning really well but now they are uncertain what to do about the weight gain, and they don't want to lose the well functioning. They feel as though they've got a child in their home for the first time. So it's a difficult situation. It's a difficult situation for us too.”*

### 4.6. Metabolic Side Effects

There was a significant discussion of “metabolic side effects”, which created decisional conflicts for participants. Parents discussed concerns of the long-term consequences based on current weight or weight gain and married this information with family histories of conditions such as diabetes. One parent also discussed a concern regarding a lack of monitoring regarding cholesterol. One prescriber discussed heart disease and cholesterol whereas others that discussed metabolic side effects in the context of weight gain focused on diabetes and glucose. 

Participants such as Youth 7 were concerned about diabetes in light of existing lab values, but his experience indicates he may not fully understand the possible risk or potential likelihood of having the “list” of side effects. 

Prescribers expressed concern regarding the side effects, and one pleaded for judicious and rational use because of limited long-term pharmacovigilance data. Prescriber 3 through encouraging the rational use of the medications also shed light on wider systems issues. Although the medications may be viewed as a “quick fix” for some in a cost-constrained system, the potential long-term implications would not be offset by immediate gratification. They also expressed concern regarding adequacy of knowledge translation efforts regarding the use of the medications. 

*Youth 7:*

*“The only other side effect I'm worrying about actually with Risperdal… Well, one other side effect is it can cause permanent diabetes. And my blood sugar levels are a little bit high since I've been taking the pill. And the other one is the inability to conceive children. That's also a possibility. And I'm worried about that one. But I mean I get this list of side effects, and I'm like, ‘Ah, what am I going to get?'.” *

*Parent 9:*

*“ So I had major, major concerns because he put on 70 pounds. … I am diabetic. And weight gain is a major factor.” *

*Parent 5:*

*“Yes, I do have a family doctor but she… I haven't talked to her. Because one of the things I noticed actually … I don't think [psychiatrist's name] had mentioned it, or I didn't actually pick it up, was [son's name] should probably actually have blood work done so he has a base to go by. Because if there's any problems with cholesterol or anything like that, he should kind of be watched. My doctor is not the greatest. You can get in to her right quick and she can give you a pad and then out you go. But he hasn't actually talked to her about anything.” *

*Focus Group Discussion*

*Prescriber 1: *

*“I am seeing younger people like in the early 20s, even in late teens, where if they have a family history of diabetes as well, it's really important to get baseline blood work because it can happen.” *

*Interview, Prescriber 2:*

*“I tend to stay away from olanzapine because I've had experience and … Well, I tend to use quetiapine. That is such a crap shoot. And I don't know … This is more like the art or the cookery, the recipe of medicine, but I've had experience with clients becoming diabetic on olanzapine.” *

*Prescriber 3:*

*“… And not just a bit of change in your blood sugar but diabetes, and changes in your cholesterol, and setting people up for heart disease later on. …  … I just don't know how widespread the caution is yet…  . Like people are like for the last 5 or 6 years have been like ‘okay, everybody, these drugs are scary'. Like, let's think. For some people, they need them, but let's be responsible. Let's think about who needs them, who doesn't. Let's think about how long they need them. Let's think about how we monitor when they are on it to make sure we don't cause them life-long problems…  But I don't know how well that knowledge has been transmitted across the board in terms of people prescribing. And I think there are serious pressures because of resource issues, not just in [location] but everywhere in mental health, that it's easier to… that drugs are more accessible in general,… cost being a factor. But it's easier to get a drug than to get therapies in general.” *

## 5. Discussion

Our qualitative study in the Canadian context is unique in that it gives insights into the lived experiences of three groups (youth, parents, and prescribers) with antipsychotic-related weight gain and related implications in the broader context of the lived experience of antipsychotics.

To our knowledge, this is the first study of this kind in Canada. The experiences of youth, parents, and prescribers were largely negative regarding weight gain and impacted body image and adherence and persistence with medications. Managing weight gain was a complex phenomenon, and various strategies were reported by participants. An overarching fear and concern related to metabolic side effects was evident in our data. 

The rising prevalence of obesity in Canadian children and adolescents has become a public health concern [43, 44]. Ball and McCargar [43] conducted a review of prevalence and risk factor literature from studies in children and adolescents. The etiology of increasingly overweight and obese children and adolescents is thought to be multifactorial including such factors related to lifestyle (e.g., limited physical activity, rising physical inactivity), eating behaviours and nutrition, and higher height and weight subsequently leading to higher BMIs with earlier maturation [43]. Medications, including psychotropics, can also cause weight gain in children and adolescents [45, 46]. Meta-analysis data ranking antipsychotic-related weight gain are available for adults [47], and mean weight changes with antipsychotics versus placebo in children up to 18 years of age show higher increases with olanzapine at 3.47 kg (95% CI 2.94, 3.99), risperidone with 1.72 kg (95% CI 1.17, 2.26), quetiapine with 1.41 kg (95% CI 1.10, 1.81), and aripiprazole at 0.85 kg (95% CI 0.58, 1.13) [15]. The experience of weight gain with antipsychotics as discussed in our study from youth, parent, and prescriber perspectives focused heavily on numeric values in pounds of weight with wide ranges of weight gain described. An inherent limitation in our study is the inability to determine if the changes self-reported by youth and parents were abnormal. We did not explicitly link weight gain with diet, physical activity, other concomitant medications, or family histories of cardiometabolic conditions. Based on the experience of some youth and parents reporting weight gains of 70 to 100 pounds, we can infer that this would be considered abnormal and its consequences in some cases would be considered significant. There was a general concern from both the prescribers and parents that the weight gain they observed, regardless of the quantity, was a negative consequence of antipsychotics for youth. 

Several anthropometric measures of body adiposity (e.g., weight, BMI, height to weight ratio, waist circumference, etc.) are available, and a standardization regarding what metrics should be used remains to be established [48]. The association of these metrics with cardiometabolic disease in adults is varied, and more research is required with the application of these metrics to long-term outcomes in children [34, 36, 43, 49–51]. The current Canadian guidelines published in 2011 on the monitoring of weight-related measures in children and adolescents taking antipsychotics recommend BMI and waist circumference measurements [52]. In our study, weight was reported as a change (i.e., increase) and in pounds. BMI and other measurements were not discussed. The Canadian guidelines were not published at the time of our interviews in 2010. The guidelines provide a good basis for clinicians to engage in monitoring practices; however, it is difficult in the context of individual patients to determine how much weight gain can be caused by individual psychotropics, the velocity at which it occurs, whether it plateaus, who is most at risk, how to manage it, and what significance is produced by the outcome given that the research in this area is limited in high quality studies of ample quantity [47, 53]. To date, studies that have examined monitoring practices show that of all parameters suggested for monitoring, weight measures are the most commonly performed [54].

A major conundrum that is apparent in the literature on the topic of antipsychotic-related weight gain in youth, or as identified in our study as “ambivalence,” emerged from our data and was aptly described by a prescriber discussing weight gain occurring in concert with much anticipated and hoped for benefits of therapy. The issue of what to do in response to weight gain is especially complex when perceived positive impacts occur. Weight gain is really one of several surrogate markers of interest in relation to cardiometabolic diseases. The potential association with chronic disease (e.g., cardiovascular and endocrine) development and related morbidity and mortality is of great importance given the significant strain on population health and economic and social welfare [55]. The issue of weight gain is complicated in clinical scenarios by the fact that more than one medication in a regimen can be the cause (e.g., lithium and olanzapine used in managing bipolar disorder) or even mitigating it (e.g., stimulants). Weight changes are also set against a backdrop of physical changes occurring at developmental stages and changes in weight that may have occurred due to illness. Further, more data is needed to provide clarity regarding weight gain and cardiometabolic risks in youth taking these medications [36]. Because of these simultaneously moving targets and need for better data, making decisions regarding the appropriate course of action can be extremely complicated for youth, families, and prescribers. How these factors are prioritized in decision making within these three groups is not well known. In our study, the concerns for metabolic side effects and weight gain were apparent and influential on behaviours. Prescribers discussed their own personal values and tacit experience with treating patients and decisional dilemmas around choice of agents with one describing it as “such a crap shoot”. Past experiences with patients developing diabetes steered one prescriber away from using the medications. Parents also described their knowledge of the side effects from what they observed and their concerns based on family histories of metabolic conditions (e.g., diabetes). Although the concern by parents was expressed, there was little resolution in their conflicted feelings as it appears that the youth and prescriber ultimately would be making decisions about weight management. 

Interestingly, the prescribers in our study tended to be focused on glucose related complications more so than cholesterol with only one prescriber discussing “heart disease” and cholesterol. The parent and youth experience of prescriber based monitoring appeared to be variable in quantity and quality. Rates of guideline concordant monitoring of antipsychotic-related cardiometabolic side effects are often poor [54]. The approach to managing and monitoring antipsychotic-related weight gain and potential cardiometabolic effects appears mainly reactive without forethought of a larger management plan for mitigating the effects.

In addition to potential physical health concerns created by obesity, negative psychosocial consequences can result from being overweight and obese [43]. Youth in our study described impacts on body image. In adults, psychotropic related weight gain has been shown to impair quality of life proportionally with weight gain and increased feeling of hopelessness and decreased self-worth [56]. Further explication of the complex relationships with antipsychotic-related weight gain in youth and its effects on quality of life, self image, and its impact on adherence in younger people is warranted [57]. 

This qualitative study demonstrates that antipsychotic-related weight gain is associated with significant physical, and mental consequences and there is a shared concern regarding these sequelae among youth, parents, and prescribers. More research is needed regarding the short- and long-term implications of weight gain and cardiometabolic effects and how the risks and benefits of treatment are managed by patients and prescribers in therapeutic decision making.

## Figures and Tables

**Table 1:** Youth participant demographics and self-reported medications and diagnoses.

Youth	Gender	Age	Medication(s) named in participant experiences	Self-reported conditions
(1) Youth 1	Female	19	Quetiapine, Strattera, Adderall	Bipolar, depressed, mood disorder, anxiety, panic attacks
(2) Antidepressant interview				
(3) Youth 2	Female	26	Olanzapine, lorazepam, risperidone, citalopram, trazodone	Psychosis, psychosis not otherwise specified, sleep disorder
(4) Youth 3	Male	25	Risperidone, olanzapine, ziprasidone, citalopram	Psychosis
(5) Youth 4	Male	24	Paxil, lithium, “a lot of different antipsychotics”	Depression, paranoid, bipolar schizophrenia, bipolar, schizo-affective, schizophrenia with bipolar
(6) Youth 5	Female	18	Quetiapine, Cipralex, clonazepam	OCD*, anxiety
(7) Youth 6	Female	21	Quetiapine, lorazepam, Risperdal, Celexa, Effexor	Schizophrenic, bipolar
(8) Youth 7	Male	24	Risperidone	Psychosis
(9) Youth 8	Male	17	Quetiapine	Paranoia
(10) Antidepressant interview				
(11) Youth 9	Male	26	Divalproex, olanzapine, clonazepam, Dilaudid	ADHD^†^, “bipolar schizophrenic”
(12) Youth 10	Female	23	Cipralex, Wellbutrin, clonazepam, risperidone	Drug-induced psychosis, Paranoid schizophrenia, ADHD^†^, anxiety
(13) Youth 11	Female	25	Lithium, olanzapine, citalopram, Ciprolex, citalopram, Wellbutrin, zopiclone	Anxiety, depression, bipolar, manic
(14) Youth 12	Female	21	Quetiapine, Zoloft, risperidone, Prozac, Paxil, lorazepam, clonazepam, diazepam, lorazepam	Borderline personality disorder
(15) Youth 13	Female	22	Lithium, risperidone, ziprasidone, Cipralex, Epival	Bipolar, psychosis
(16) Youth 14	Male	20	Risperidone, Dexedrine, Ritalin, Wellbutrin	ADHD^†^, ADD^‡^, seizure disorder, autism
(17) Youth 15	Male	13	Biphentin, risperidone	ADHD^†^, anger problem, violent behaviour

*OCD: Obsessive compulsive disorder.

^†^ADHD: attention deficit hyperactivity disorder.

^‡^ADD: attention deficit disorder.

## List of Brand Name Medications (Alphabetical) Discussed by Participants and Their Respective Generic Names

Adderall: Dextroamphetamine and levoamphetamine mixed salts
Biphentin: Methylphenidate
Cipralex: Escitalopram
Dexedrine: Dextroamphetamine
Dilaudid: Hydromorphone
Epival: Divalproex sodium
Invega: Paliperidone
Paxil: Paroxetine
Prozac: Fluoxetine
Risperdal: Risperidone
Ritalin: Methylphenidate
Seroquel: Quetiapine
Strattera: Atomoxetine
Wellbutrin: Bupropion
Zoloft: Sertraline.
